# How Does Policy Support Affect the Behavior and Effectiveness of Domestic Waste Classification? The Mediating Role of Environmental Protection Perception

**DOI:** 10.3390/ijerph20032427

**Published:** 2023-01-30

**Authors:** Ya Huang, Zhangbao Zhong

**Affiliations:** 1 College of Economics & Management, Huazhong Agricultural University, Wuhan 430070, China; 2 Research Center for Rural Social Construction & Management, Huazhong Agricultural University, Wuhan 430070, China

**Keywords:** domestic waste classification, classification behavior, classification effectiveness, policy support, environmental protection perception

## Abstract

Waste classification is the key initiative to solve environmental pollution and achieve resource recycling, environmental improvement, and high-quality development in rural areas. Under the perspective of “external support–internal drive”, this paper adopts the survey data of 2628 rural residents in Jiangsu province to analyze the influence and spatial effect of policy support (PS) on the behavior and effectiveness of rural residents’ domestic waste separation and the mediating effect of environmental protection perception using the PSM and Sobel test. The study found that: (1) PS has a significant positive effect on the governance of the domestic residents’ waste classification in rural areas. The variation in classification behavior (BWC) is more obvious than classification effectiveness (EWC). (2) PS has a significant difference in the positive influence on waste separation by regions. Specifically, the enhancement effect of PS on BWC decreases from south to north, and the enhancement effect on EWC is more obvious in southern Jiangsu than in central Jiangsu, but not significant in northern Jiangsu. (3) The mediating effect indicates that PS promotes the enhancement of BWC and EWC by strengthening farmers’ environmental protection perceptions, and the effect of EWC enhancement is more obvious. Drawing on the results of this paper, the government should improve the policy support system for waste classification, strengthen environmental protection-related policy publicity and knowledge popularization, stimulate the endogenous motivation of rural residents, narrow the regional environmental governance gap, and promote green and sustainable development in rural areas.

## 1. Introduction

The global energy crisis and environmental pollution problems are becoming increasingly serious, and how to achieve resource recycling and ecological sustainability has become the exploratory direction for countries worldwide. As an essential component of sustainable resource management, separating household garbage among rural residents is a powerful step towards improving the quality of the human environment and the living environment of rural residents [1]. It is also an important way to build ecological civilization and ensure public health and safety in rural areas. However, since the policy of waste classification was implemented, the overall effect of rural household waste classification in China has been relatively inefficient, and the treatment efficiency needs to be increased continuously [2]. In particular, the total amount and variety of domestic waste in rural areas is enormous, and about 40% of them have not been treated effectively [3], which has caused multiple malfunctions in the production and life of rural residents [4]. In order to solve these problems, the central and local governments have issued a series of policy documents to provide policy support for improving the rural environment. The No. 1 document of the Central Government in 2022 emphasizes promoting rural waste classification and treatment continuously and establishing a long-term and orderly classification mechanism (http://www.moa.gov.cn/govpublic/FZJHS/202203/t20220301_6389886.htm accessed on 28 January 2023). The 20th Communist Party of China National Congress stated, “China will further promote the prevention and control of environmental pollution, enhance the effectiveness of waste classification and promote ecological sustainability” (http://www.gov.cn/xinwen/2022-10/25/content_5721685.htm accessed on 28 January 2023). It indicates that the prevention and governance of China’s environmental pollution have entered a new stage of improving quality and efficiency. In order to enhance the effectiveness of rural waste separation and mobilize rural residents to participate in waste separation, local governments have enacted various reward and punishment systems and carried out policy promotion, which has achieved specific results [5]. However, the effects of waste separation policy formulation and implementation are varied widely [6]. Therefore, the question is whether policy support could effectively promote rural residents’ participation in waste separation? What is the overall effectiveness of waste segregation management? Does the enactment and implementation of policy provisions effectively improve the effectiveness of waste separation? Is there a significant difference in the effect of policy support in different regions? Answering these questions is closely related to solving environmental pollution problems and promoting eco-green development.

Studies have found that external factors influence rural domestic waste sorting behaviors and effectiveness in the form of governmental instruments or organizational support [7,8], as well as internal factors such as individual perceptions [9]. Based on the external environment perspective, a portion of scholars discussed the relationship between rural residents’ domestic waste sorting behavior and the institutions of village autonomy and environmental remediation [10], different forms of incentives [11], rewards and punishments [6], and information interventions [3]. Further discussion reveals that information interventions can significantly improve the effectiveness of classification governance for village residents, and the effects of different information interventions differed remarkably [3,12]. As evidenced by overseas studies, insufficient policy support and inadequate policy implementation systems can lead to chaos in waste governance [13]. In China’s rural social development, implementing government-led institutional design is vital in promoting rural environmental governance [14]. On the other hand, some scholars have also assessed the elements influencing classification behavior at the individual psychological dimension. Internal motivations research emphasizes the influential role of individual emotion and subjective perceptions of personality. It was found that residents’ perceptions of waste classification [5,15,16], environmental pollution [17], institutional trust [18], and choice of off-farm employment [19] significantly and positively influenced farmers’ willingness to engage in the waste separation. 

This excellent research has provided scientific evidence for formulating environmental policies and improving the effectiveness of waste separation, but there are some limitations. (1) In terms of research dimensions, most studies focused on whether waste separation policies could achieve constraints and guidance on the behavior of rural residents, while few studies considered the influence and regional differences of institutional measures on the effectiveness of waste separation. (2) In terms of research content, most articles investigate the relationship between waste separation policies with the behavior of government and rural households. However, the efficiency of the institutional constraints and endogenous incentives models for rural environmental governance are both very limited at present. One of the important reasons is that the environmental awareness of rural residents is relatively poor [20]. Rural residents’ level of environmental perception influenced the overall effectiveness of waste separation to a large extent. Ostrom’s theory of autonomous governance finds that as producers of household waste and beneficiaries of the treatment effect, motivating residents to actively participate in domestic waste sorting is fundamental to completely solving rural environmental governance problems [21,22]. Therefore, it is especially essential to build a synergistic governance model combining external support and internal motivation. (3) From the perspective of the research mechanism, studies focused on the impacts of policy support and environmental perception on waste separation behavior and effectiveness, but there are fewer studies on how these elements work effectively. 

In conclusion, policy support and rural residents‘ environmental protection perception may affect waste separation behavior and effectiveness. Meanwhile, environmental governance policies are an essential factor influencing rural residents‘ environmental protection perception [10]. So, environmental perception may play an important role in the pathway that policy support affects sorting behavior and effectiveness. Therefore, the purpose of this study is to investigate the influence effect and mechanism of policy support on waste classification behavior and effectiveness in rural areas. Specifically, under the perspective of external constraints and internal drive for co-governance, this study uses field survey data of rural residents in Jiangsu province of China to investigate the impacts and regional variations of policy support on the classification behavior and effectiveness, and verify the mediating role of environmental perception. This paper aims to provide optimal suggestions and practical references for improving the effect of rural household waste sorting and promoting the overall improvement of the rural environment.

This article is organized as follows. Section 1 proposes the background, research review, and research purposes through an introduction of this paper. Section 2 presents the literature review and research hypothesis; Section 3 shows the model, data source, and variable selection; Section 4 provides the empirical results. Section 5 discusses the empirical tests and policy recommendations. Finally, Section 6 states the conclusions and limitations.

## 2. Literature Review and Research Hypothesis

### 2.1. Literature Review

Along with implementing many policies such as the improvement of the living environment in rural areas, scholars have widely discussed the governance of rural domestic waste classification. The existing studies have focused on the classification mode [4,22,23], willingness to classify [5,10,18,20,24], classification behavior [5,6,25], and classification effect [2,3]. However, there are few studies in the existing literature on the mechanisms influencing the behavior and effectiveness of domestic waste classification. As the main subject and direct beneficiary of rural environmental governance, the progress and results of waste separation directly affect their happiness index. Farmers‘ participation in waste separation is both a rational economic decision and an environmental protection behavior with public awareness. Currently, waste segregation governance as a quasi-public good is in the exploratory and practical stage in rural areas. Various supports from the government and rural communities are important forces to promote waste sorting policy implementation and enhance sorting effectiveness [26]. Numerous international findings on waste separation have shown that policy support significantly impacts the environmentally beneficial behaviors of rural residents [27]. In addition, waste sorting policies could significantly enhance the subjective perceptions and attitudes of rural residents toward waste sorting [28]. Therefore, farmers‘ participation in waste separation governance is not only based on policy factors but also influenced by farmers’ environmental perceptions.

The impact of policy support on the governance of rural residents’ waste sorting has received attention from scholars. Factors such as institutional norms and environmental policies could influence farmers’ environmental behavior [24] and inadequate policy norms are considered to be an important reason for the slight effectiveness of waste sorting in China [29]. Some scholars believe that waste separation governance should be carried out by establishing perfect institutional norms. At the same time, the policy measures should be enforced strictly with the support of external policies, and the enforcement of material punishment should be strengthened for violations of the laws and regulations [23,30], combined with informal institutions such as township rules and regulations [14], to guide rural residents to form green and healthy production and living habits [31]. Therefore, the behavior of rural residents in waste separation is an autonomous decision-making behavior under a certain policy environment. In the context of building an ecologically livable rural society, the relationship between policy support and waste classification behavior and effectiveness needs to be further explored.

In addition, psychological factors such as environmental perceptions could have some influence on farmers’ environmental behavior [32]. Rural residents are less aware of environmental protection, have lower ecological perception [33], and lack knowledge about the hazards of environmental pollution and waste separation [34,35], whereas individuals with a higher level of environmental awareness are more willing to adopt green and pro-environmental behaviors [36]. Therefore, the lack of environmental protection cognition might discourage farmers to participate in waste separation to a certain extent, thus affecting the effectiveness of waste separation. Moreover, the new institutional economics school of thought argues that behavior occurs in the institutional environment, which influences individual preferences and self-perceptions by providing actors with cognitive templates essential for activities to affect individual behavioral choices [37]. Because of this, this paper incorporates environmental perceptions into the framework of policy support waste sorting behavior and effectiveness research to reveal the mediating role of environmental protection perceptions in the pathway of policy support affecting sorting behavior and effectiveness.

### 2.2. Research Hypothesis

#### 2.2.1. Policy Support and Rural Residents’ Domestic Waste Classification Behavior and Effectiveness

The first issue is the influence of policy support on waste classification behavior. The formulation and implementation of various policies and regulations will not only intervene effectively in farmers’ activities of waste sorting governance but also establish a favorable institutional environment to promote farmers’ positive expectations of waste sorting governance and increase their initiative to participate in environmental governance [38,39]. The development and implementation of environmental policies are important factors influencing rural environmental governance. Secondly, the effect of policy support on the effectiveness of waste separation is significant. The practical experience of rural household waste sorting in China over the years shows that measures such as propaganda guidance and spiritual encouragement have little effect; the effect of waste sorting by incentives alone is very limited. The key to the effectiveness of rural waste sorting is properly implementing rules and regulations. Policy regulation is a crucial element of influence to stimulate farmers to participate in household waste sorting and improve the effectiveness of environmental governance [10]. Rural residents know about domestic waste through various policies, and information intervention could significantly improve the effectiveness of domestic waste sorting and governance for rural residents [3]. Factors such as the compulsion and diversity of environmental policies and the continuity of policy output are important paths to promote the transformation of policy advantages into environmental governance effectiveness [40]. Therefore, this paper argues that perfect and effective policy measures could positively guide farmers’ participation in waste sorting behavior and promote the overall improvement of sorting effectiveness. With the above analysis, the research hypothesis is as follows:

**Hypothesis** **1a** **(H1a).** *Policy support has a notable improvement in waste separation behavior.*

**Hypothesis** **1b** **(H1b).** *Policy support has a remarkable impact on waste separation effectiveness.*

#### 2.2.2. Policy Support, Environmental Perceptions and Rural Residents’ Behavior and Effectiveness in Waste Classification

According to the Theory of Planned Behavior and Behavioral Economics, the institutional environment plays an important role in shaping individuals’ cognition, while individual behaviors often occur based on their level of cognition [10]. Farmers with higher levels of environmental cognition tend to have more environmental awareness and are more motivated to adopt environmental behavior [5]. Specifically, farmers’ perceived attitudes toward the hazards of environmental pollution and the benefits of waste sorting would induce an intrinsic drive for individuals to participate in environmental protection, thus motivating farmers to engage in various forms of environmental protection activities on their initiative [41]. Individuals could motivate environmental-protection behaviors when they psychologically perceive the severity of environmental pollution [42]. In addition, an important mechanism that policy support influences rural residents’ domestic waste sorting is environmental protection perception. The increase in farmers’ knowledge of waste sorting could have a positive contribution to sorting behavior and sorting effectiveness [17]. It has been shown that environmental policies have an important impact on rural residents’ environmental protection perceptions [43]. Environmental policies could increase farmers’ understanding of waste separation and strengthen separation skills. With the guidance of policies, farmers will actively participate in environmental treatment based on the importance of the responsibility, and perceived benefits of, classifying waste. This will accelerate the environmental governance effectiveness [44]. Therefore, this paper concludes that rural residents with higher environmental perceptions have a strong intrinsic drive for waste sorting, and that the sorting knowledge and skills provided by policy support have a positive contribution to waste sorting, which enhances the overall effect of waste sorting. Due to the previous analysis, the research hypothesis of this paper is as follows:

**Hypothesis** **2a** **(H2a).** *Environmental protection perceptions are intermediate in policy support affecting the classification behavior.*

**Hypothesis** **2b** **(H2b).** *Environmental protection perceptions are intermediate in policy support affecting the classification effectiveness.*

**Figure 1:**
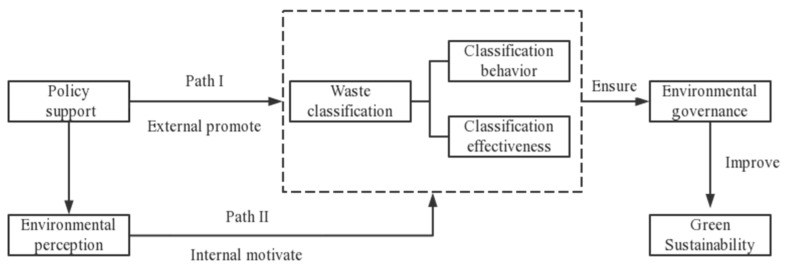
Theoretical analysis model.

Figure 1 demonstrates the path of policy support on waste behavior and effectiveness.

## 3. Materials and Methods

### 3.1. Data Sources

All the data used in this article are from a survey conducted by Nanjing Agricultural University in Jiangsu Province in 2020 (CLES). Jiangsu Province’s innovative and practical model of classified management of rural domestic waste provides an example of the construction of beautiful villages across the country. The survey was conducted using stratified and random sampling methods of “city-county-township-village-rural residents”, and 26 counties were randomly selected from 13 cities in Jiangsu Province, China. Moreover, the research sample was chosen randomly from 50 rural households in each village. The investigators carried out face-to-face surveys with respondents in rural areas by distributing questionnaires, and the sample involved 52 villages in 52 townships. At last, we obtained 2628 valid questionnaires. The questionnaire includes the willingness and behavior of household garbage separation for rural residents, an evaluation of the effectiveness of domestic waste separation for rural inhabitants, relevant government policies and measures, rural residents’ perception of environmental protection, and essential domestic information. 

Table 1 shows the basic characteristics of the questionnaire data. Firstly, the respondents were mainly men (69.82%) who were over 45 years old (92.01%) and did not have a political identity (70.24%), and most respondents were over 60 years old (55.06%). Secondly, the respondents’ average years of education was 6.89 years, and nearly half (44.36%) of the farmers had only received an elementary school education or less, indicating that the average education level of the respondents was generally relatively weak, which may affect the rural residents’ perception of environmental policies and their motivation to participate in waste separation. Furthermore, in terms of the village institutional environment, the percentage of village committees holding environmental improvement meetings is 82.18%, which may affect the effectiveness of environmental protection policies to some extent. The survey results, such as the age and years of education of the farmers interviewed, demonstrate that the survey sample is representative, similar to the statistics of Jiangsu Province.

### 3.2. Model Selection

#### 3.2.1. Basic Regression Model

This article focuses on the effect of policy support on the behavior and effectiveness of rural waste separation. As the garbage sorting behavior is a two-classification variable, and the garbage classification effect is an ordinal categorical variable, referring to the research of related scholars [6], this paper selects the binary probit model and ordered probit model (Oprobit) for an empirical framework. The specific model is as follows:

(1) $$Behavior_{i}=\alpha_{0}+\alpha_{1}Measures_{i}+\alpha_{2}X_{i}+\mu_{i}$$

(2) $$Effectiveness_{i}=\beta_{0}+\beta_{1}Measures_{i}+\beta_{2}X_{i}+\mu_{i}$$

Expression (1) indicates the effects of policy support on the behavior of waste classification, and expression (2) indicates the impact of policy support on the waste classification effect. Where $Behavior_{i}$ is the waste sorting behavior of the observed rural resident i, $Effectiveness_{i}$ is the waste sorting effect of the observed rural resident i, $Measures_{i}$ is policy support, $X_{i}$ is a series of control variables, and $\mu_{i}$ is a stochastic disturbance item.

#### 3.2.2. Intermediary Effects Model

Secondly, to estimate the intermediary effect of environmental protection perception between policy support and the behavior and effectiveness of waste classification regarding the relevant literature [45], this paper sets up a mediating effect model as follows:

(3) $$Y_{i}=\lambda_{0}+\lambda_{1}Measures_{i}+\lambda_{2}X_{i}+\mu_{i}$$

(4) $$Perception_{i}=\eta_{0}+\eta_{1}Measures_{i}+\eta_{2}X_{i}+\mu_{i}$$

(5) $$Y_{i}=\gamma_{0}+\gamma_{1}Measures_{i}+\gamma_{2}Perception_{i}+\gamma_{3}X_{i}+\varphi_{i}$$

where $\lambda_{1}$ reflects the total effect of policy support on the behavior and effect of village waste management of the i farmers, $\eta_{1}$ indicates the effect of policy support on the mediating variable environmental protection perceptions, the mediating effect of policy support $\eta_{1}\gamma_{2}$ and the share of mediating effect is $\eta_{1}\gamma_{2}$/$\lambda_{1}$.

### 3.3. Variable Description

#### 3.3.1. Dependent Variable

This article’s dependent variables are the behavior and effectiveness of waste classification for rural inhabitants. At first, the behavior of rural residents’ domestic waste classification (BWC) is a binary dummy variable. In our survey, we use the question “Do you classify your domestic waste?” to measure the BWC in the questionnaire. Next, the effectiveness of waste classification for rural inhabitants’ households (EWC) is an ordered multi-categorical variable. In the survey, the question is set as follows: “Do you agree that the domestic waste classification has a positive effect on the improvement of the rural environment?” This question asks farmers to rate the current system’s effectiveness in classifying household waste in rural areas. The answer options are “1 = Totally disagree, 2 = Disagree, 3 = Neutral, 4 = Agree, 5 = Agree completely”. The higher the value, the more positive the farmers’ evaluation of the EWC and the more effective the governance.

#### 3.3.2. Core Independent Variable

Policy support (PS) is the core explanatory variable. The question “Has the government implemented various policies such as incentives and penalties for classifying rural waste?” measures the PS in governing domestic waste classification in rural areas (Table 2).

#### 3.3.3. Mediating Variable

Environmental protection perception (EPP) is the mediating variable in this article. This paper measures EPP through farmers’ judgments of the current rural domestic waste classification level (Table 3). In order to have a more comprehensive and specific understanding of farmers’ psychological perceptions of rural residents’ domestic garbage sorting, combine the actual circumstances of the study and refer to the research results of related scholars [5,15,17]. We examine farmers’ perceptions of the policy on household waste classification, environmental protection, and their behavior towards household waste separation and management in their villages.

#### 3.3.4. Control Variables

It has been found that farmers’ characteristics (gender, age, education year), household endowments (household size, social network, political identity, household expenditure), farmers’ institutional trust, political participation, and village meetings affect farmers’ environmental governance behavior [10]. The variables mentioned above were used as control variables in our research. The specific definition and descriptive statistics of the variables are shown in Table 4.

## 4. Empirical Results

### 4.1. Multicollinearity Test

Based on PS and EPP, our research uses the probit and Oprobit model to analyze the influence mechanism of the BWC and EWC. Firstly, the KMO statistic calculated through SPSS 22.0 was more significant than 0.6, and Bartlett’s test was passed at the 1% significance level, indicating that the variable measures have validity and reliability. Secondly, we apply the variance inflation factor (VIF) to test for multicollinearity among the explanatory variables in this article. The findings showed that the maximum VIF is 1.47, the average is 1.20, and the tolerance ranges are from 0.666 to 0.946. Consequently, there is no collinearity problem between the explanatory variables (Table 5).

### 4.2. Results of the Basic Regression Model

The research uses the probit and Oprobit models for empirical analysis by Stata15.1 software (tataCorp LLC, 4905 Lakeway Drive, College Station, Texas, TX, USA). At the same time, we measured the variables’ marginal effects to investigate further the impact of the variables on the BWC and EWC. Table 6 reports the estimation results of the models, and it can be seen that the results of the chi-square tests of the model regressions all passed the 1% significance level test.

#### 4.2.1. Impact of PS on the BWC

Table 6 presents the empirical results of the baseline regression model. We can see that PS significantly affects the elevation of BWC at the 1% significance level. In addition, the marginal effect demonstrates that the BWC can increase by 32.7% with the PS of rural inhabitants increasing by one unit. That is to say, the stronger the PS for a complete solution to the environmental challenges caused by household waste, the higher of possibility for rural residents to participate in classifying. It shows that PS is the key to motivating farmers to participate in classifying actively. Studies have also shown that improving the mechanism of rural waste management, enhancing investment in infrastructure, and building the rule of law can increase farmers’ motivation to participate in environmental governance and eliminate bottlenecks in waste classification [46]. Hypothesis 1a is verified.

#### 4.2.2. Impact of Policy Support on the EWC

Moreover, the results in Table 6 show that PS significantly impacts the EWC at a 1% significance level. Furthermore, the marginal effect demonstrates that the PS of rural residents increases by one unit, and the EWC probability of “very effective” can increase by 10.8 percentage points. It indicates that the more substantial PS by government-imposed incentives and penalties for waste classification provides a strong policy constraint on the household waste classification for rural inhabitants. It is beneficial to improve the evaluation of the EWC for rural residents. Participating in rural domestic waste classification can promote the potential for non-participating households to segregate their waste through a “network of acquaintances”. In a word, government incentives and penalties are a robust regulatory system for rural residents. Hypothesis 1b is verified here.

In addition, for control variables, EY impact rural residents’ BWC and EWC positively at the 1% significance level. The marginal effect shows that with a one-year increase in years of education of rural inhabitants, the BWC could increase by 0.9%, and the EWC could increase by 1.4%. In other words, the higher the literacy level of the residents, the more environmental protection awareness they have; they are more willing to participate in the classification of domestic waste actively. That is to say, the longer the farmers have been educated, the more knowledge they have, the easier it is to understand and accept the household waste separation, and the higher their willingness to improve their household living environment [47]. Moreover, social network positively affect the BWC and EWC in rural areas at the 1% significance level. The more extensive the social network of farmers, the stronger their ability to obtain information, and the deeper their understanding of policy norms and environmental protection, the easier it is to engage in the classification of household waste. Institutional trust also had a positive impact on the BWC and EWC in rural areas at a 1% significance level. The reason may be that rural residents believe in government decision-making. The higher the degree of trust in the system, the higher the incentives for farmers to engage in rural governance actions, and the greater the probability of abiding by the behavior of classified management of rural garbage, which is consistent with existing scholars [48]. Farmers’ participation in village politics can deepen the knowledge and implementation of village environmental governance, thus increasing the likelihood of participating in rural domestic waste classification [25,49]. On the contrary, the age of rural residents negatively impacts waste separation at a 1% significance level. The reason may be that EPP among the elderly is relatively low, and it is more challenging to implement waste classification policies. There was no statistically significant effect of other control variables on the BWC and EWC among rural residents.

### 4.3. Heterogeneity Analysis: Spatial Effects

To further analyze the characteristics of the impact of PS on the BWC and EWC of rural residents’ household, we divided the samples into three sub-samples according to the regional distribution: northern Jiangsu, central Jiangsu, and southern Jiangsu (North Jiangsu includes Xuzhou, Lianyungang, Suqian Municipality, Huai’an, and Yancheng; Central Jiangsu includes Yangzhou, Taizhou, and Nantong; South Jiangsu includes Nanjing, Suzhou, Wuxi, Changzhou, and Zhenjiang). The results in Table 7 show that the overall trend of the impact of SP on governance behaviors and effect evaluation is south of Jiangsu > middle of Jiangsu > north of Jiangsu. Moreover, the results of a further marginal effects analysis suggest that the probability of PS positively impacting the BWC is 29.79% higher in the southern Jiangsu region and 18.83% higher than in the central Jiangsu region. Furthermore, the results of the EWC show that the impact of PS has a positive significance at the 5% level in the central Jiangsu region and a 1% level in the Southern Jiangsu areas but insignificant in the northern Jiangsu region. That is to say, the enhancement effect of PS on rural residents’ domestic waste separation and disposal shows noticeable regional heterogeneity. The possible reasons are, firstly, the regional development is unbalanced. Northern Jiangsu is a traditional agricultural growing area, which is far away from the Shanghai metropolitan circle, and the regional comprehensive development is quite a bit lower. The infrastructure for waste separation and disposal is insufficient in rural areas, thus the policy implementation environment is worse; secondly, the rural residents in the backward areas have weak environmental awareness and knowledge of waste separation, which makes it more difficult to carry out waste separation; additionally, the government in the lower development has not enough ability to comprehend and implement the policy, thus the appeal to guide rural residents to participate in waste separation is insufficient.

### 4.4. Mediating Effects of Environmental Protection Perceptions

Taking into account the method proposed by relevant scholars [50], we examined the mediating effect of EPP in the pathway of PS to influence the BWC and EWC. The results in Table 8 demonstrate the estimated coefficients of PS. The statistically estimated coefficients of PS in models (1) and (4) are significantly positive, which means PS plays a positive role in the BWC and EWC. Then, model (2) shows that PS contributes positively to EPP. Moreover, the estimated coefficients of PS and EPP in models (3) and (5) are both significantly positive, indicating that EPP plays a part in the mediating effect. It is known by calculation that the intermediary effect of EPP in the impact of PS on the classified governance of rural domestic waste is 22.30%, and the intermediary effect in the EWC is 35.43%. It indicates that about 22.30% and 35.43% of the positive impact of PS on the BWC and EWC for rural residents is mediated by EPP. In addition, the robustness test conducted in this paper by the Sobel test and bootstrap statistic shows that the mediating effect is significant at the 1% level, and the 95% confidence interval of the estimated parameters does not contain 0, suggesting that the estimation results are robust. Hypotheses 2a and 2b are therefore verified.

### 4.5. Robustness Tests

To further test the robustness of the previous analysis results, this paper employs a quasi-natural experiment to verify the differences between PS and the BWC and EWC of rural residents. Specifically, we used Propensity Score Matching (PSM) to compare the differences between the control group (with policy support) and the treatment group (without policy support). Furthermore, we fit the average treatment effect (ATT) of PS in the waste classification of rural residents. After sample matching smoothness tests, we used three methods of Nearest Neighbor Matching (1−4 matching), Caliper Matching (radius = 0.01), and Kernel Matching (broadband = 0.06) to estimate the ATT of PS. 

The results in Table 9 show that the pseudo R2 value significantly decreased from 0.034 before matching to 0.002 under the different matching methods. The standard bias for each variable was significantly reduced to below 10%. Moreover, the probability that PS could enhance the BWC ranges from 0.329 to 0.337, and the average governance effect of PS for the EWC ranges from 0.165 to 0.188. The differences in individual characteristics between the experimental and treatment group samples were partially eliminated, implying that the individual differences between the experimental and control groups affecting the action of rural household waste segregation management were insignificant, except for the difference in the presence or absence of PS. The results of Nearest Neighbor Matching, Caliper Matching, and Kernel Matching were generally consistent, and ATT passed the significance test, implying that we resolved the endogeneity of the variable ‘self-selection.’ That is to say, PS significantly promotes the classification and management of rural household waste. Therefore, the significance of the results obtained from the PSM estimation is coherent with the regression results of the baseline regression model, demonstrating that the results of the above experimental analysis are relatively robust.

## 5. Discussion and Policy Implication

### 5.1. Discussion

The separation and treatment of rural residents’ household waste is the key and challenging point of environmental governance and a critical task in constructing ecological civilization and implementing a rural revitalization strategy. Rural inhabitants are not only the implementers of various waste classification policies but also the direct beneficiaries of the effects of waste classification. The national governments at all levels have developed and implemented various policy norms to guide and incentivize rural residents to actively participate in waste classification to promote high-quality rural development and meet people’s needs for a better life. In this context, we constructed a theoretical analysis framework of external support and internal drive. We conducted an empirical analysis by combining survey data from rural areas in Jiangsu, China. Compared with the willingness studies currently conducted by most scholars, this paper examines the current influencing factors, paths of action, and regional differences in rural household garbage governance from the viewpoint of the behavior and effects of waste sorting governance, making the conclusions more realistic and instructive. The study of the BWC and EWC based on PS and EPP could provide concrete policy recommendations for improving institutional arrangements, enhancing farmers’ ecological perceptions, and narrowing regional disparities. Actual studies in other eastern provinces could refer to the research design of this paper to reduce investigation costs, and analyses in central and western regions can refer to the research ideas and modeling methods of this paper. 

### 5.2. Policy Implications

The paper draws the following policy implications in light of the above discussions.

(1) Improve the design of the rural waste separation institutions to promote them to be effectively implemented in rural areas. Due to the wide gap between urban and rural areas in China, including the weak economic development and inadequate waste treatment facilities, the design of policies and regulations should apply the basic characteristics of rural communities and residents in order to establish more scientific institutional norms. Secondly, optimizing the criteria for waste separation and improving the feasibility of policy adoption. Rural residents are generally less educated, and the requirements and standards for garbage classification should take rural residents as the main subjects of practice to promote the effective transformation of policy norms into effective concrete practices. In addition, establish various forms of reward and punishment institutions. Encourage rural households to participate in waste separation through a community “honor roll” and “point system”, and impose material punishment on those who violate the system seriously to implement the policies and regulations strictly.
(2) Strengthen the level of environmental protection recognition of rural residents and enhance the initiative of waste separation participation. On the one hand, rural communities could carry out activities such as policy interpretation, technical training, and knowledge popularization of garbage classification in combination with diversified policy publicity channels including village meetings and agricultural training to improve farmers’ awareness of ecological protection, environmental pollution and the benefits of treatment, stimulate farmers’ main motivation, and cultivate rural residents to develop a green and healthy production and lifestyle. On the other hand, we could combine online and offline publicity methods and carry out publicity activities in various forms such as TV, cell phones, wall posters, and advertisements to promote farmers’ multi−faceted and all−round understanding of waste separation.
(3) Optimize the diversified support path of rural household waste classification governance and narrow the regional development gap. First, make up for the shortcomings in rural areas. Policy support should be changed from universal to focused, according to the actual regional development, or “tailor-made”, especially for less developed areas or ecologically cultured areas. It is necessary to make policy innovations according to local conditions and strengthen financial support, infrastructure construction, and talent training. Second, give full play to the advantages of rural resources. From a single type of project input to comprehensive project implementation, focus on technical input, personnel division of labor and service management, and other professional system construction in areas with good resources, accelerate the development of the resource nation and marketization of rural household waste, and further promote the transformation of policy advantages into governance effectiveness.

## 6. Conclusions and Limitations

### 6.1. Conclusions

Establishing a sustainable and effective policy support system is an important driver for the sustainable ecological development of China, and strengthening environmental perceptions through PS is a critical driver for the treatment of rural household waste classification. The government’s practical guidance and farmers’ active participation are the critical paths to achieving the effectiveness of rural residents’ household waste classification. Previous studies on the influencing factors of waste separation have neglected to explore this mechanism. Therefore, in order to examine the effectiveness of PS in the governance of rural household waste classification, this paper investigates the influence of policy norms on farmers’ participation in waste sorting and the improvement of classification effectiveness, discusses how this influence works, and whether this impact is different in various regions. This study analyzed the influence and mechanism of PS on the BWC and EWC of rural residents. We used micro-survey data in Jiangsu Province of China, and the PSM and Mediating Effect models to analyze the empirical evidence. 

Based on these findings, this paper draws the following conclusions. At first, the baseline regression shows that PS has a significant positive effect on rural household BWC and EWC. Particularly, the positive influence of PS on BWC is more pronounced than the EWC. Secondly, heterogeneity regression analysis shows significant regional differences in the impacts of PS on BWC and EWC. Specifically, the spatial effect of PS on BWC decreased from south to north, while the improvement effect on EWC is not significant in northern Jiangsu, but exerts greater effectiveness in southern Jiangsu than in central Jiangsu. Furthermore, the mediating effect shows that PS promotes BWC and EWC improvement by strengthening farmers’ EPP, and the variation in EWC is more significant. That means that part of PS’s positive effect on waste classification is achieved through farmers’ EPP. At the same time, the mediating effect of farmers’ EPP in the influence path of the EWC is more substantial, indicating that farmers’ initiative is essential in improving the EWC in the rural area. 

### 6.2. Limitations and Future Research

In addition, this study has several limitations that can provide directions for future research. First, the measurement of PS is based on an overall policy examination in this paper, and there is a lack of objective data on specific policy measures such as financial, material, and technical support. Future research could discuss how different policy support approaches and degrees affect the BWC and EWC. Furthermore, this research is based on cross-sectional data, which cannot explain the time effect between PS and waste separation. Along with the comprehensive promotion of the waste separation process in China, future studies could analyze the dynamic trends of policy effectiveness by combining national multi-period data. In addition, it is worth noting that 53.01% of the farmers in the data sample think that the current effect of rural household waste classification and governance needs to be improved. Therefore, future research could focus on exploring the integration of a “recycling network” and a “waste collection and transportation system network” in order to promote the resource nation and marketization of rural waste, and explore the spillover effects of waste separation on the improvement of rural residents’ happiness and the sustainable development of rural communities.

## Figures and Tables

**Table 1:** Basic information of questionnaire data.

Variable	Category	Sample	Percentage/%	Variable	Category	Sample	Percentage/%
Sex	Male	1835	69.82	Political Identity	Yes	779	29.68
	Female	793	30.18		No	1846	70.32
Years of Education	0	358	13.63	Village Meeting	Yes	2093	82.18
	≤6	807	30.73		No	454	17.82
	>6–9	999	38.04	Age	≤45	210	7.99
	>9–12	350	13.33		>45–60	971	36.95
	>12	112	4.27		>60	1447	55.06

Note: Political identity means whether the farm household is a CPC member. Village meeting means does the village committee hold environmental improvement meetings.

**Table 2:** Core variables’ definition and descriptive statistics.

Variable	Mean	S.D.	Min	Max	Description
BWC	0.485	0.500	0	1	The behavior of waste classification for rural inhabitants
EWC	4.213	0.991	1	5	The effectiveness of waste classification for rural inhabitants
PS	0.202	0.402	0	1	Policies support of rural household waste classification

**Table 3:** Intermediary variable indicator and weight.

Variable	Dimension	Indicator	Weight	Mean	S.D.
EPP	Policy Awareness	Policy perceptions of garbage classification and habitat improvement and policy advocacy policy publicity	0.40	0.485	0.500
	Environmental Perception	Evaluation of habitat environment; evaluation of ecological civilization	0.33	4.213	0.991
	Behavioral Perception	Self-evaluation and others’ evaluation of environmental protection behavior	0.27	1.568	0.876

**Table 4:** Variable definition and descriptive statistics.

Variable	Mean	S.D.	Min	Max	Description
Sex	0.701	0.458	0	1	Gender of farm household respondents: male = 1, female = 0
Age	61.046	11.329	17	90	Age of farm household respondents (years)
EY	6.900	3.948	0	18	Years of education of farm respondents (years)
FS	3.226	1.664	1	11	The family population of household respondents (person)
PI	0.297	0.457	0	1	The family political attributes of respondents: Party = 1, Non-party = 0
SN	4.977	17.073	0	100	Number of people to turn to in case of hardship (person)
lnHE	10.248	0.852	7.090	14.367	Total household expenditure in 2019 taken as a logarithm
VM	0.822	0.383	0	1	Does the village committee hold environmental improvement meetings
IT	4.035	0.808	1	5	Respondents’ level of trust in village officials
PP	1.568	0.876	1	3	Farmers participate in village election voting

**Table 5:** Multicollinearity test.

Variable Name	VIF	Tolerance	Variable Name	VIF	Tolerance	Variable Name	VIF	Tolerance	Variable Name	VIF	Tolerance
EWC	1.05	0.9547	Sex	1.16	0.8608	FS	1.25	0.7991	LnHE	1.33	0.7530
PS	1.12	0.8963	Age	1.47	0.6790	PI	1.15	0.8711	VM	1.16	0.8638
PP	1.23	0.8135	EY	1.46	0.6851	SN	1.05	0.9515	IT	1.07	0.9348

**Table 6:** Impact of PS on the BWC and EWC.

Variable	Classification Behavior		Classification Effectiveness					
	BWC	Marginal Effects	EWC	Marginal Effects				
				Almost No Effect	Little Effect	Attitude Neutrality	More Effective	Extremely Effective
PS	0.920 ***	0.327 ***	0.281 ***	−0.026 ***	−0.006 ***	−0.030 ***	−0.045 ***	0.108 ***
	(0.071)	(0.023)	(0.060)	(0.006)	(0.002)	(0.007)	(0.010)	(0.023)
Sex	−0.060	−0.021	0.046	−0.004	−0.001	−0.005	−0.007	0.018
	(0.063)	(0.022)	(0.055)	(0.005)	(0.001)	(0.006)	(0.009)	(0.021)
Age	−0.012 ***	−0.004 ***	−0.005 *	0.000 *	0.000 *	0.001 *	0.001 *	−0.002 *
	(0.003)	(0.001)	(0.002)	(0.000)	(0.000)	(0.000)	(0.000)	(0.001)
EY	0.024 ***	0.009 ***	0.037 ***	−0.003 ***	−0.001 ***	−0.004 ***	−0.006 ***	0.014 ***
	(0.008)	(0.003)	(0.007)	(0.001)	(0.000)	(0.001)	(0.001)	(0.003)
FS	−0.036 **	−0.013 **	−0.022	0.002	0.000	0.002	0.004	−0.009
	(0.018)	(0.006)	(0.015)	(0.001)	(0.000)	(0.002)	(0.002)	(0.006)
PI	0.006	0.002	0.066	−0.006	−0.001	−0.007	−0.011	0.025
	(0.062)	(0.022)	(0.054)	(0.005)	(0.001)	(0.006)	(0.009)	(0.021)
SN	0.014 ***	0.005 ***	0.002	−0.000	−0.000	−0.000	−0.000	0.001
	(0.004)	(0.001)	(0.002)	(0.000)	(0.000)	(0.000)	(0.000)	(0.001)
LnHE	0.046	0.016	0.094 ***	−0.009 ***	−0.002 ***	−0.010 ***	−0.015 ***	0.036 ***
	(0.036)	(0.013)	(0.031)	(0.003)	(0.001)	(0.003)	(0.005)	(0.012)
VM	0.040	0.014	−0.038	0.004	0.001	0.004	0.006	−0.015
	(0.075)	(0.027)	(0.064)	(0.006)	(0.001)	(0.007)	(0.010)	(0.025)
IT	0.099 ***	0.012 ***	0.145 ***	−0.014 ***	−0.003 ***	−0.015 ***	−0.023 ***	0.055 ***
	(0.034)	(0.012)	(0.030)	(0.003)	(0.001)	(0.003)	(0.005)	(0.011)
PP	−0.125 ***	−0.044 ***	0.000	−0.000	−0.000	−0.000	−0.000	0.000
	(0.034)	(0.012)	(0.029)	(0.003)	(0.001)	(0.003)	(0.005)	(0.011)
Sample	2425	2425	2423	2423	2423	2423	2423	2423
LR chi2	339.94 ***		148.28 ***					
Pr2	0.1011		0.0269					

Note: *, **, and ***, respectively, pass the significance test at the statistical levels of 10%, 5%, and 1%. Standard errors are in parentheses.

**Table 7:** Spatial effects of policy support: marginal effects.

Variables	Classification Behavior			Classification Effectiveness		
	Northern Jiangsu	Central Jiangsu	Southern Jiangsu	Northern Jiangsu	Central Jiangsu	Southern Jiangsu
PS	0.282 ***	0.308 ***	0.366 ***	0.033	0.130 **	0.177 ***
	(0.035)	(0.049)	(0.037)	(0.033)	(0.053)	(0.037)
Control variables	YES	YES	YES	YES	YES	YES
Sample	931	565	929	933	562	928
LR chi2	110.57 ***	105.63 ***	156.07 ***	67.49 ***	48.66 ***	53.23 ***
Pseudo R2	0.0866	0.1360	0.1228	0.0303	0.0370	0.0287

Note: ** and ***, respectively, pass the significance test at the statistical levels of 5% and 1%. Standard errors are in parentheses.

**Table 8:** Intermediary effect of environmental protection perceptions.

Variables	Classification Behavior			Classification Effectiveness	
	BWC	EPP	BWC	EWC	EWC
	(1)	(2)	(3)	(4)	(5)
PS	0.920 ***	0.244 ***	0.759 ***	0.281 ***	0.169 ***
	(0.071)	(0.027)	(0.074)	(0.060)	(0.062)
EPP	−	−	0.841 ***	−	0.408 ***
			(0.058)		(0.046)
Control variables	YES	YES	YES	YES	YES
Sample	2425	2390	2385	2423	2384
LR chi2/F	339.94 ***	68.61 ***	547.19 ***	148.28 ***	215.25 ***
Pseudo R2/Adjust R2	0.1011	0.2374	0.1656	0.0269	0.0397

Note: ***, respectively, pass the significance test at the statistical level of 1%. Standard errors are in parentheses.

**Table 9:** Impact of policy support on the BWC and EWC: PSM model.

Variables	Classification Behavior			Classification Effectiveness		
	Nearest Neighbor Matching	Caliper Matching	Nuclear Matching	Nearest Neighbor Matching	Caliper Matching	Nuclear Matching
IS	0.337 *** (0.026)	0.332 *** (0.023)	0.329 *** (0.023)	0.188 ** (0.057)	0.170 *** (0.053)	0.165 *** (0.052)
%bias	Below 10% after matching					
Pseudo R2	Before matching → after matching: 0.034 → 0.002					
Sample	2425			2423		

Note: *** and **, respectively, pass the significance test at the statistical levels of 1% and 5%. Standard errors are in parentheses.

## Data Availability

The datasets used and analyzed in this study are available upon request from Nanjing Agricultural University and the corresponding author with the permission of Nanjing Agricultural University.
